# Surgical outcomes of spinal fusion for osteoporotic thoracolumbar vertebral fractures in patients with Parkinson’s disease: what is the impact of Parkinson’s disease on surgical outcome?

**DOI:** 10.1186/s12891-019-2473-8

**Published:** 2019-03-09

**Authors:** Kei Watanabe, Keiichi Katsumi, Masayuki Ohashi, Yohei Shibuya, Tomohiro Izumi, Toru Hirano, Naoto Endo, Takashi Kaito, Tomoya Yamashita, Hiroyasu Fujiwara, Yukitaka Nagamoto, Yuji Matsuoka, Hidekazu Suzuki, Hirosuke Nishimura, Hidetomi Terai, Koji Tamai, Atsushi Tagami, Syuta Yamada, Shinji Adachi, Toshitaka Yoshii, Shuta Ushio, Katsumi Harimaya, Kenichi Kawaguchi, Nobuhiko Yokoyama, Hidekazu Oishi, Toshiro Doi, Atsushi Kimura, Hirokazu Inoue, Gen Inoue, Masayuki Miyagi, Wataru Saito, Atsushi Nakano, Daisuke Sakai, Tadashi Nukaga, Shota Ikegami, Masayuki Shimizu, Toshimasa Futatsugi, Seiji Ohtori, Takeo Furuya, Sumihisa Orita, Shiro Imagama, Kei Ando, Kazuyoshi Kobayashi, Katsuhito Kiyasu, Hideki Murakami, Katsuhito Yoshioka, Shoji Seki, Michio Hongo, Kenichiro Kakutani, Takashi Yurube, Yasuchika Aoki, Masashi Oshima, Masahiko Takahata, Akira Iwata, Hirooki Endo, Tetsuya Abe, Toshinori Tsukanishi, Kazuyoshi Nakanishi, Kota Watanabe, Tomohiro Hikata, Satoshi Suzuki, Norihiro Isogai, Eijiro Okada, Haruki Funao, Seiji Ueda, Yuta Shiono, Kenya Nojiri, Naobumi Hosogane, Ken Ishii

**Affiliations:** 1 Department of Orthopedic Surgery, Niigata University Medical and Dental General Hospital, 1-757 Asahimachi Dori, Chuo-ku, Niigata City, Niigata 951-8510 Japan; 2 Department of Orthopaedic Surgery, Osaka University, 2-2 Yamadaoka, Suita City, Osaka 565-0871 Japan; 3 Department of Orthopaedic Surgery, Tokyo Medical University, 6-1-1 Shinjuku, Shinjuku-ku, Tokyo 160-8402 Japan; 4 Department of Orthopaedic Surgery, Osaka City University, 1-4-3 Asahimachi, Abeno-ku, Osaka 545-8585 Japan; 5 Department of Orthopaedic Surgery, Nagasaki University, 1-7-1 Sakamoto, Nagasaki City, Nagasaki 852-8501 Japan; 6 Department of Orthopaedic Surgery, Tokyo Medical and Dental University, 1-5-45 Yushima, Bunkyo-ku, Tokyo 113-8519 Japan; 7 Department of Orthopaedic Surgery, Kyushu University, 3-1-1 Maidashi, Higashi-ku, Fukuoka City, 812-8582 Japan; 8 Department of Orthopaedic Surgery, Jichi Medical University, 3311-1 Yakushiji, Shimotsuke City, Tochigi 329-0498 Japan; 9 Department of Orthopaedic Surgery, Kitasato University, 1-15-1 Kitasato, Minami-ku, Sagamihara City, Kanagawa 252-0374 Japan; 10 Department of Orthopaedic Surgery, Osaka Medical College, 2-7 Daigakumachi, Takatsuki City, Osaka 569-0801 Japan; 11 Department of Orthopaedic Surgery, Tokai University, 143 Shimokasuya, Isehara City, Kanagawa 259-1193 Japan; 12 Department of Orthopaedic Surgery, Shinshu University, 3-1-1, Asahi, Matsumoto City, Nagano 390-8621 Japan; 13 Department of Orthopaedic Surgery, Chiba University, 1-8-1 Inohana, Chuo-ku, Chiba City, Chiba 260-8670 Japan; 14 Department of Orthopaedic Surgery, Nagoya University, 65 Tsurumai-cho, Showa-ku, Nagoya City, Aichi 466-8560 Japan; 15 Department of Orthopaedic Surgery, Kochi University, Oko-cho Kohasu, Nankoku City, Kochi 783-8505 Japan; 16 Department of Orthopaedic Surgery, Kanazawa University, 13-1 Takaramachi, Kanazawa City, Ishikawa 920-8641 Japan; 17 Department of Orthopaedic Surgery, University of Toyama, 2630 Sugitani, Toyama City, Toyama 930-0194 Japan; 18 Department of Orthopaedic Surgery, Akita University, 1-1-1 Hondo, Akita City, Akita 010-8543 Japan; 19 Department of Orthopaedic Surgery, Kobe University, 7-5-1 Kusunoki-cho, chuou-ku, Kobe City, Hyogo 650-0017 Japan; 20 Department of Orthopaedic Surgery, Eastern Chiba Medical Center, 3-6-2 Okayamadai, Togane City, Chiba 283-8686 Japan; 21 Department of Orthopaedic Surgery, Nihon University Itabashi Hospital, 30-1 Oyaguchikamicho, Itabashi-ku, Tokyo 173-8610 Japan; 22 Department of Orthopaedic Surgery, Hokkaido University, North-15, West-7, Kita-ku, Sapporo City, Hokkaido 060-8638 Japan; 23 Department of Orthopaedic Surgery, Iwate Medical University, 19-1 Uchimaru, Morioka City, Iwate 020-8505 Japan; 24 Department of Orthopaedic Surgery, University of Tsukuba, 1-1-1 Tennodai, Tsukuba City, Ibaraki 305-8577 Japan; 25 Department of Orthopaedic Surgery, Hiroshima University, 1-2-3 Kasumi, Minami-ku, Hiroshima City, Hiroshima 734-8551 Japan; 26 Department of Orthopaedic Surgery, National Defense Medical College, 3-2 Namiki, Tokorozawa City, Saitama 359-8513 Japan; 27 Department of Orthopaedic Surgery, Keio University School of Medicine, 35 Shinanomachi, Shinjuku-ku, Tokyo 160-8582 Japan; 28 Department of Orthopaedic Surgery, International University of Health and Welfare School of Medicine, Mita, Minato-ku, Tokyo 108-8329 Japan

**Keywords:** Parkinson’s disease, Osteoporosis, Vertebral fracture, Spinal fusion, Thoracolumbar spine, Visual analogue scale, Japanese orthopedic association score, Outcome, Perioperative complication, Kyphosis

## Abstract

**Background:**

To date, there have been little published data on surgical outcomes for patients with PD with thoracolumbar OVF. We conducted a retrospective multicenter study of registry data to investigate the outcomes of fusion surgery for patients with Parkinson’s disease (PD) with osteoporotic vertebral fracture (OVF) in the thoracolumbar junction.

**Methods:**

Retrospectively registered data were collected from 27 universities and their affiliated hospitals in Japan. In total, 26 patients with PD (mean age, 76 years; 3 men and 23 women) with thoracolumbar OVF who underwent spinal fusion with a minimum of 2 years of follow-up were included (PD group). Surgical invasion, perioperative complications, radiographic sagittal alignment, mechanical failure (MF) related to instrumentation, and clinical outcomes were evaluated. A control group of 296 non-PD patients (non-PD group) matched for age, sex, distribution of surgical procedures, number of fused segments, and follow-up period were used for comparison.

**Results:**

The PD group showed higher rates of perioperative complications (*p* < 0.01) and frequency of delirium than the non-PD group (p < 0.01). There were no significant differences in the degree of kyphosis correction, frequency of MF, visual analog scale of the symptoms, and improvement according to the Japanese Orthopaedic Association scoring system between the two groups. However, the PD group showed a higher proportion of non-ambulators and dependent ambulators with walkers at the final follow-up (p < 0.01).

**Conclusions:**

A similar surgical strategy can be applicable to patients with PD with OVF in the thoracolumbar junction. However, physicians should pay extra attention to intensive perioperative care to prevent various adverse events and implement a rehabilitation regimen to regain walking ability.

**Electronic supplementary material:**

The online version of this article (10.1186/s12891-019-2473-8) contains supplementary material, which is available to authorized users.

## Background

Parkinson’s disease (PD) is an age-related, neurodegenerative disorder with a prevalence that is increasing as the population ages. It is characterized by motor- and various non-motor symptoms, which, in particular, increases the risk of falls and consequent fragility fractures. A large epidemiological study of community dwelling elderly women reported that people with PD were more likely to sustain a fracture than their peers (hazard ratio, 2.2; 95% confidence interval, 1.6–3.1). [1] This has been caused by a higher chance of both falls and reduced bone mineral density (BMD) in patients with PD. [2] Reduced BMD is common, and can be typically diagnosed using dual X-ray absorptiometry imaging. [3] A recent study of 186 patients with PD at the early stage demonstrated that 11.8 and 41.4% of patients were diagnosed as osteoporosis (T-score less than − 2.5), and osteopenia (T-score between − 1 and − 2.5), respectively. [4] In addition, reduced BMD can be caused by immobility, vitamin D deficiency, use of dopaminergic treatments, and reduced nutritional intake in patients with PD.

Osteoporotic vertebral fracture (OVF) is the most common fragility fracture and frequently causes back pain, neurological symptoms, and spinal deformity. Thoracolumbar OVF is a common spinal disorder in elderly patients, [5, 6] and the number of patients with thoracolumbar OVF undergoing spinal fusion has been increasing in our aging society. Consequently, a large variety of surgical fusion techniques have been used to treat OVF including anterior spinal fusion (ASF) [7, 8]; posterior spinal fusion alone (PSF) [9, 10]; combined anterior and posterior spinal fusion (APSF) [11]; posterior 3 column osteotomy (3CO), including shortening osteotomy [12, 13] or vertebral column resection [14]; and vertebroplasty with posterior spinal fusion (VP + PSF) [13, 15–17]. However, there is little published data on surgical outcomes of spinal fusion for patients with PD with thoracolumbar OVF.

We hypothesized that patients with PD with thoracolumbar OVF suffered from poorer surgical outcomes, including frequency of perioperative complications and quality of life, compared to non-PD patients. To evaluate this hypothesis, we conducted a retrospective review of a multicenter database of patients with OVF in the thoracolumbar spine to clarify the effectiveness and associated problems of fusion surgery for patients with PD.

## Methods

This study was reviewed and approved by the institutional review board of all institutions involved. The study was performed by JASA (Japan Association of Spine Surgeons with Ambition) using a retrospective analysis of patients with OVF treated by spinal fusion surgery at 27 university hospitals and their affiliated hospitals. A total of 26 patients with PD (PD group), including 3 men and 22 women, were identified based on the following inclusion criteria: 1) OVF in the thoracolumbar spine (from T10 to L2); 2) existence of neurological impairment, including motor deficit or neuralgia in the lower extremity; 3) underwent instrumented spinal fusion concomitant with autologous bone grafting (excluding stand-alone vertebroplasty and kyphoplasty); and 4) a minimum of 2 years of follow-up after surgery. The diagnosis of PD was based on the United Kingdom Parkinson’s Disease Society Brain Bank Criteria. [18] The average age and body mass index (BMI) at the time of surgery were 75.7 years (range, 67–89 years) and 21.8 kg/m^2^ (range, 14.2–34.9 kg/m^2^), respectively. The collapsed vertebral levels were T11, T12, L1, and L2 in 3, 8, 11, and 4 patients, respectively. The surgical procedures consisted of 4 typical techniques: PSF (*n* = 3), APSF (*n* = 2), 3CO (*n* = 9), and VP + PSF (*n* = 12), and the mean number of fused segments was 4.1 segments (range, 2–8 segments). The mean PD duration was 60.0 ± 50.6 months (range, 0–168 months), and the Hoehn and Yahr stage [19] was stage 1, 2, 3, and 4 in 2, 6, 13, and 5 patients, respectively. Demographic data are shown in Table 1.

**Table 1:** Comparison of demographic data between the 2 groups

	PD group	Non-PD group	*p* value
N of patients	26	296	–
Age at operation (years) median, [IQR]	76.0 [8.0]	75.0 [10.8]	0.3343
Sex [male/female] (N of patients)	3/23	66/230	0.1999
BMI (kg/m^2^) median, [IQR]	22.1 [6.3]	22.5 [4.7]	0.1600
Vertebral level (N of patients)	T11: 3	T10: 16	0.6112
	T12: 8	T11: 25	
	L1: 11	T12: 116	
	L2: 4	L1: 103	
		L2: 36	
Surgical procedure (N of patients)	APSF: 2	ASF: 19	0.7176
	PSF: 3	APSF: 27	
	3CO: 9	PSF: 37	
	VP + PSF: 12	3CO: 84 VP + PSF: 129	
Number of fused segment (segment) median, [IQR]	4.0 [2.0]	4.0 [2.0]	0.9534
BMD YAM (%) median, [IQR]	73.0 [25.0]	69.0 [19.8]	0.9295
Number of patients with existing vertebral fracture [fx/no fx] (N of patients)	8/18	108/188	0.7338
Number of comorbidity (disease) median, [IQR]	1.0 [0.0]	1.0 [1.0]	< 0.0001
Follow-up period (month) median, [IQR]	37.0 [19.0]	44.0 [28.0]	0.1787

*Abbreviation*: *N* number, *IQR* interquartile range, *BMI* body mass index, *ASF* anterior spinal fusion, *APSF* combined anterior and posterior spinal fusion, *PSF* posterior spinal fusion, *3CO* 3 column osteotomy, *VP + PSF* vertebroplasty with PSF, *BMD* bone mineral density, *YAM* young adult mean, *fx* fracture

The control group comprised 296 non-PD patients with thoracolumbar OVF (non-PD group) whose data were retrieved from the same database (Table 1); there were no statistically significant differences with respect to age, sex, BMI, distribution of collapsed vertebral levels, distribution of surgical procedures, number of fused segments, and follow-up period between the PD and non-PD groups (*p* > 0.05 for all comparisons). The outcome measures were compared between the 2 study groups.

### Surgical procedure

The surgical procedures comprised various instrumentations or bone grafting techniques used in the retrospective multicenter database. The ASF surgical procedure was performed using a rod or plate system with an iliac or rib bone strut graft or metal cage. The APSF surgical procedure was a combination of ASF using an iliac or fibula strut graft and PSF using a pedicle screw and rod system. The PSF surgical procedure was performed using a pedicle screw and rod system, occasionally using laminar hooks in the uppermost or lowermost instrumented vertebra. The 3CO surgical procedure consisted of PSF as described above and vertebral column resection with reconstruction using a metal cage or eggshell shortening osteotomy through the posterior approach only. For VP + PSF, the surgical procedure consisted of PSF as described above and VP using hydroxyapatite blocks or paste performed via a transpedicular approach.

### Evaluation

**Fig. 1:**
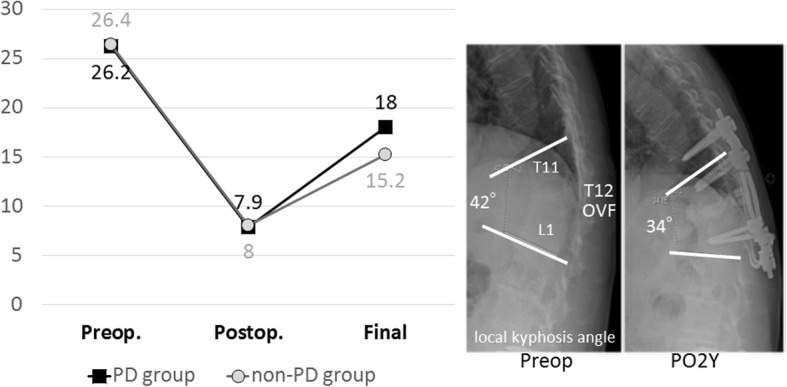
Line graph showing the change in the mean local kyphosis angle preoperatively, postoperatively, and at final follow-up. Both groups showed significant correction between the before surgery and the final follow-up (*p* < 0.05). PD, Parkinson’s disease

Surgical invasion, radiographic sagittal alignment, mechanical failure (MF), and clinical outcomes were evaluated from medical charts, plain radiographs, and computed tomography images. The evaluation of surgical invasion included the operation time, intraoperative blood loss, and perioperative complications. Radiographic sagittal alignment included the local kyphosis angle on the lateral view of plain radiographs measured between the upper endplate of the uninvolved vertebra above the affected level and the lower endplate of the uninvolved vertebra below the affected level using the Cobb method (Fig. 1). The evaluation of mechanical failure included the presence of pedicle screw pull-out, cage migration, fracture of the uppermost or lowermost instrumented vertebra, hook dislodgement, and rod fracture. Clinical outcomes were evaluated using the visual analog scale (VAS; ranging from 0 [no symptoms] to 100 [worst symptoms]) for lower back pain and lower extremity pain or numbness; the Japanese Orthopaedic Association Scoring system ([JOA score], ranging from 0 [worst condition] to 15 [best condition]) (Additional file 1); walking ability using the following grading system: grade 1, independent walking; 2, dependent walking with a cane; 3, dependent walking with walker; and 4, unable to walk (requiring a wheelchair); and occurrence of subsequent vertebral fracture. The rate of improvement in both lower back pain and lower extremity pain was assessed with the JOA score using Hirabayashi’s method [20] as follows: ([postsurgical score - presurgical score] / [15 - presurgical score] × 100).

### Statistical analysis

All analyses were performed using StatView-J 5.0 software (Abacus Concepts, Berkeley, CA). The changes in investigated parameters before and after surgery were evaluated using the nonparametric Wilcoxon signed-rank test. The changes in continuous and discrete variables between the two groups were compared using the nonparametric Mann-Whitney U test and the chi-squared test, respectively. *P* < 0.05 was considered to be statistically significant in all analyses.

## Results

The results are summarized in Table 2.

**Table 2:** Comparison of the outcome variables data between the 2 groups

	PD group	Non-PD group	p value
Operation time (min.) median, [IQR]	214.0 [100.0]	237.0 [130.5]	0.4193
Intraoperative blood loss (ml) median, [IQR]	450.0 [627.0]	402.0 [575.5]	0.2761
Perioperative complication [complication/ no complication] (N of patients)	10/16	45/251	0.0063
Local kyphosis angle (°) median, [IQR]			
Preop.	27.0 [21.0]	26.0 [19.0]	0.9965
Postop.	6.0 [18.0]	8.0 [13.4]	0.8072
Final	18.7 [18.0]	14.0 [18.0]	0.3357
Amount of kyphosis correction	10.8 [26.1]	10.0 [16.0]	0.6611
Mechanical failure (%)	26.9	17.9	0.7464
JOA score improvement rate (%) median, [IQR]	50.0 [38.5]	53.8 [39.2]	0.1074
Walking ability (N of patients)			
Preop.	Grade1: 2	Grade1: 16	0.1030
	Grade 2: 1	Grade 2: 44	
	Grade 3: 4	Grade 3: 86	
	Grade 4: 19	Grade 4: 150	
Final	Grade1: 3	Grade1: 114	0.0007
	Grade 2: 5	Grade 2: 92	
	Grade 3: 12	Grade 3: 70	
	Grade 4: 6	Grade 4: 19	
Subsequent vertebral fracture (%)	38.4	35.1	0.7338

*Abbreviation*: *N* number, *IQR* interquartile range, *JOA* score, Japanese Orthopaedic Association scoring system

### Surgical invasion

There were no significant differences in the operation time and intraoperative blood loss between the 2 groups (p > 0.05 for both comparisons). The PD group showed a higher rate of perioperative complications (odds ratio 3.48; 95% CI 1.488–8.168, *p* = 0.0060) and frequency of delirium than the non-PD group (PD group: 23.1%, non-PD group: 3.4%)(odds ratio 8.58; 95% CI 2.83–26.009, *p* < 0.0001) (Table 3).

**Table 3:** Details of perioperative complications

Perioperative complication	PD group (N of patients)	Non-PD group (N of patients)
Overall [incidence]	10 [38.5%]	45 [15.2%]
Intraoperative complication		
Surgical site infection	0	7
Neurological deficit	1	6
Dural tear	0	5
Epidural hematoma	0	3
Massive hemorrhage (> 5000 ml)	0	1
Postoperative complication		
Delirium	6	10
Cardiac disease	0	4
Gastrointestinal disease	0	4
Deep venous thrombosis	1	2
Urinary tract infection	1	0
Pneumonia	0	2
Electrolyte abnormality	0	1
Decubitus	1	0

*Abbreviation*: *N* number

### Radiographic sagittal alignment

Regarding the correction of the local kyphosis angle after surgery, both groups showed significant correction between the before surgery and the final follow-up (*p* < 0.05 for both comparisons) (Fig. 1). There were no significant differences in the degree of kyphosis correction between the groups (p > 0.05).

### MF

In the PD group, 8 mechanical failures (26.9%) were identified. There were no significant differences in the frequency of mechanical failures between the two groups (Table 4).

**Table 4:** Details of mechanical failures

Mechanical failure	PD group (N of patients)	Non-PD group (N of patients)
Overall [incidence]	7 [26.9%]	53 [17.9%]
Pedicle/vertebral screw pull-out	4	24
Intervertebral cage migration	0	9
Uppermost vertebral fracture	2	11
Lowermost vertebral fracture	1	4
Hook dislodgement	0	3
Rod fracture	0	2

*Abbreviation*: *N* number

### Clinical outcome

**Fig. 2:**
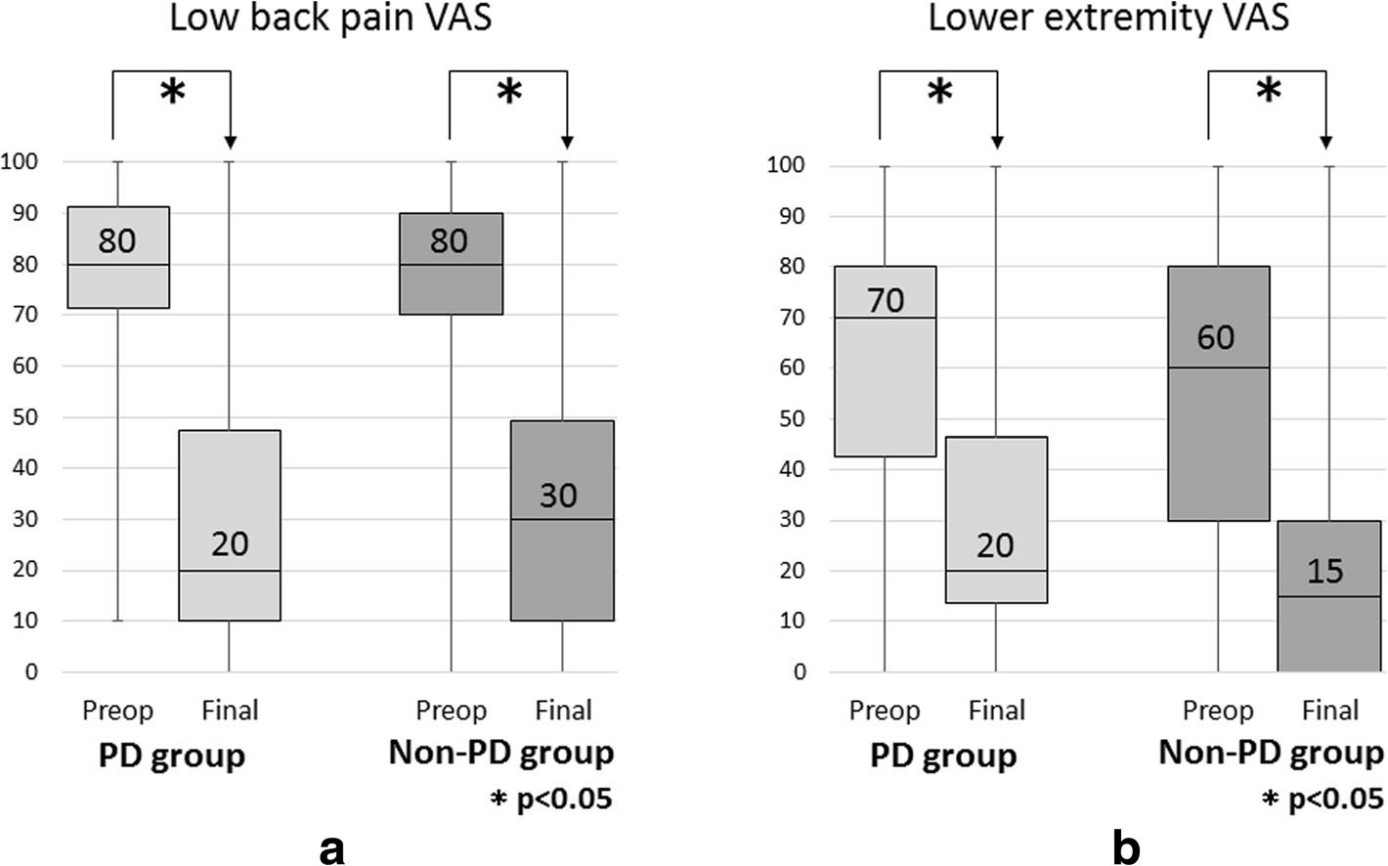
**a** Box and whisker plot showing the mean lower back pain VAS preoperatively and at the final follow-up. **b** Box and whisker plot showing the mean lower extremity pain VAS preoperatively and at the final follow-up. VAS, visual analog scale

**Fig. 3:**
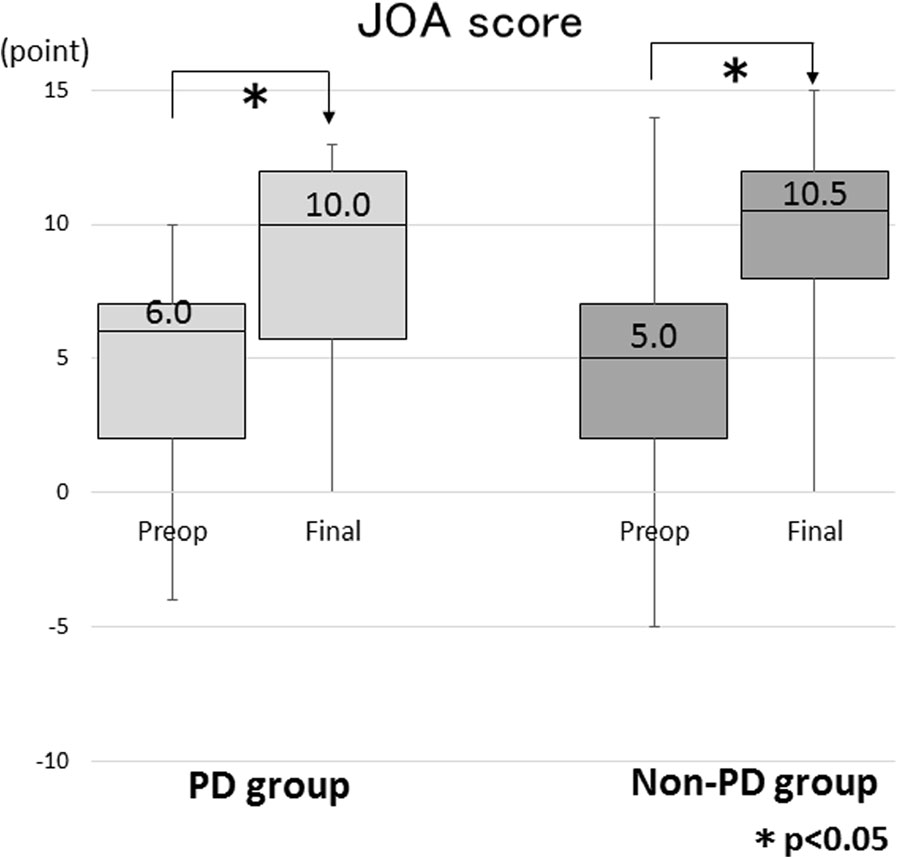
Box and whisker plot showing the mean JOA scores preoperatively and at the final follow-up. JOA, Japanese Orthopaedic Association Scoring system

Regarding the severity of neurological symptoms according to the VAS, both groups demonstrated significant improvement in lower back pain and lower extremity pain at the final follow-up (p < 0.05 for all comparisons) (Fig. 2). There were no significant differences in the VAS preoperatively and at the final follow-up between the groups. Both groups demonstrated significant improvement in the JOA score (p < 0.05 for all comparisons) (Fig. 3), and there was no significant difference in the improvement rate between the groups. There were no significant differences in the walking ability grade preoperatively; however, the PD group showed a higher proportion of patients in grades 3 and 4 at the final follow-up (odds ratio 3.788; 95% CI 1.719–8.347, *p* = 0.0007). Overall, 114 patients (35.4%) sustained a subsequent vertebral fracture and there were no significant differences in the incidence between the groups.

## Discussion

In the present study, patients with PD unexpectedly demonstrated acceptable and similar clinical outcomes compared to non-PD patients, including surgical invasion, local kyphosis correction, frequency of instrumentation-related MF, severity of symptoms, and JOA score. On the other hand, patients with PD demonstrated a higher rate of perioperative complications and inferior walking ability after surgery due to characteristic physical conditions related to PD itself.

### Frequency of perioperative complications

According to a large, national insurance database, PD was significantly associated with an increased risk for major medical complications (adjusted OR, 1.22; 95% CI, 1.11–1.34) including myocardial infarction, acute renal failure, pulmonary embolism, cerebrovascular accidents, and pneumonia following thoracolumbar fusion surgery. [21] According to another large, nationwide inpatient database, PD was a significant predictor of major postoperative complications (OR, 1.74; 95% CI, 1.37–2.22) including surgical site infection, sepsis, pulmonary embolism, respiratory complications, cardiac events, stroke, and renal failure following spine surgery. [22] In addition, postoperative delirium was more common in patients with PD (30.3%) than in the controls (4.3%), [22] which was in agreement with the present study. Postoperative delirium is a common complication of surgical procedures in the elderly, [23] and acute delirium increases morbidity and mortality leading to prolonged hospitalization. [24, 25] Therefore, physicians should be aware of the various adverse events that may occur, especially due to interruption of anti-parkinsonism drugs following spine surgery. Moreover, a noteworthy finding is that despite the relatively higher risk of potentially fatal parkinsonism-hyperpyrexia syndrome, [26] no such cases occurred in the present study. Needless to say, the establishment of a partnership between orthopedic surgeons and neurologists is essential for perioperative care, and early intervention against adverse events is desirable.

### Surgical strategy for patients with PD and OVF in the thoracolumbar junction

Thoracolumbar OVF is a common spinal disorder in elderly patients, [5, 6] which frequently causes neurological symptoms including spinal cord or cauda equina impairment. Based on previous reports, a consensus has emerged that delayed neurological impairment following OVF is primarily caused by instability of the fracture site rather than mechanical neural compression by ectopic bony fragments. [9, 10, 15] Based on the previous studies, patients with PD have higher chance of postoperative complications and unintended revision surgeries after spinal fusion. Additionally, surgically treated patients with PD tend to have poorer outcomes and lower fusion rates, especially in patients who undergo multi-level fusion. [27, 28] A consensus has emerged that long-segment corrective fusion surgery tends to be necessary for global sagittal malalignment, owing to the progressively stooped posture as PD progresses, and the risk of unfavorable biomechanics related to a long lever arm at the lumbosacral junction. [29] Although various surgical procedures have provided acceptable outcomes for thoracolumbar OVF, we hypothesized that patients with PD and thoracolumbar OVF may have poorer surgical outcomes. In the present study, they surprisingly showed acceptable outcomes as assessed by several indicators including frequency of perioperative complications, amount of kyphosis correction, and improvement of the VAS and JOA score. With regard to the walking ability, patients with PD had a higher proportion of non-ambulators and dependent ambulators with walkers, which might be caused by the diminished baseline physical capacity due to PD itself. Therefore, the results of the present study can conclude that the same conventionally used surgical indications are applicable to PD patients with OVF in the thoracolumbar junction.

There are some limitations of this study. First, the study design was retrospective, and the study was based on data review, which did not allow us to evaluate the severity of preoperative vertebral collapse, surgical details, such as choice of approach, use of supplemental anchors, and concomitant decompression procedures, and global spinal alignment. Second, selection bias could not be avoided due to different indications for non-PD and PD patients based on the various motor or non-motor symptoms associated with PD. Third, we could evaluate the PD status according to the simple 5-grade classification, but could not evaluate the severity of motor- or non-motor symptoms associated with PD. Therefore, a prospective study with a larger sample size that provides detailed specific symptoms on PD must be conducted to elucidate the effect of PD on surgical outcomes in patients with OVF. Despite these limitations, this study presents the largest case series evaluating the surgical outcomes in patients with PD and OVF in the thoracolumbar junction; the number of such patients is currently increasing due to unprecedented aging of the population.

## Conclusion

Spinal fusion for patients with PD and OVF in the thoracolumbar junction resulted in good radiological and symptomatic improvement, except for frequency of perioperative complications and functional improvement of walking ability, compared to non-PD patients. Moreover, they were similar with regard to prevalence of instrumentation-related MF and subsequent vertebral fracture. The results of this study imply that same conventionally used surgical strategy can be applicable for patients with PD and OVF in the thoracolumbar junction. However, multidisciplinary, intensive perioperative care must be provided by the orthopedic surgeons and neurologists in unison to prevent various adverse events and a rehabilitation regimen implemented to regain the patients’ walking status before the OVF-related injury.

## Additional file

Additional file 1: The assessment Scale Proposed by the Japanese Orthopaedic Association. The Japanese Orthopaedic Association Scoring system (JOA score) consists of 2 categories (subjective and objective symptoms), ranging from 0 (worst condition) to 15 (best condition). (DOCX 22 kb)

## Abbreviations

ASF: anterior spinal fusion
BMD: bone mineral density
JOA: Japanese Orthopaedic Association
OVF: osteoporotic vertebral fracture
PD: Parkinson’s disease
PSF: posterior spinal fusion
VAS: Visual Analog Scale
