# Supplementary material for: Surgical outcomes of spinal fusion for osteoporotic thoracolumbar vertebral fractures in patients with Parkinson’s disease: what is the impact of Parkinson’s disease on surgical outcome?
Source: BMC Musculoskelet Disord. 2019 Mar 9;20:103. doi: 10.1186/s12891-019-2473-8 (PMC6408814; doi:10.1186/s12891-019-2473-8)
Supplement: Supplementary file 1 — The assessment Scale Proposed by the Japanese Orthopaedic Association. The Japanese Orthopaedic Association Scoring system (JOA score) consists of 2 categories (subjective and objective symptoms), ranging from 0 (worst condition) to 15 (best condition). (DOCX 22 kb) [file 12891_2019_2473_MOESM1_ESM.docx]

Additional file 1 The assessment Scale Proposed by the Japanese Orthopaedic Association

Score

Subjective symptoms (9 points)

Low back pain

None 3

Occational mild 2

Always present or occational severe 1

Always severe 0

Leg pain/tingling

None 3

Occational mild 2

Always present or occational severe 1

Always severe 0

Ability to walk

Normal 3

~500m 2

500m~ 1

At most 100m 0

Objective findings (6 points)

SLR (including hamstring tightness)

Normal 2

30-70 degree 1

<30 degree 0

Sensory abnormality

Normal 2

Mild disturbance 1

Distinct 0

MMT

Normal 2

Slight decrease 1

Marked decrease 0

Total score 15 points

MMT indicates manual muscle test; SLR, straight leg raising test.
